# Effect of non-surgical, non-pharmacological weight loss interventions in patients who are obese prior to hip and knee arthroplasty surgery: a rapid review

**DOI:** 10.1186/s13643-015-0107-2

**Published:** 2015-09-27

**Authors:** Michelle Lui, C. Allyson Jones, Marie D. Westby

**Affiliations:** Department of Occupational Therapy, Surrey Memorial Hospital, 13750 96 Avenue, Surrey, BC V3V 1Z2 Canada; Department of Physical Therapy, University of Alberta, 8205 114 Street, 2-50 Corbett Hall, Edmonton, AB T6G 2G4 Canada; School of Public Health, University of Alberta, c/o Arthritis Research Canada, 5591 No. 3 Road, Richmond, BC V6X 2C7 Canada

**Keywords:** Rapid review, Total hip arthroplasty, Total knee arthroplasty, Weight management, Weight loss, Surgical outcomes, Post-operative complications

## Abstract

**Background:**

Of the more than 104,000 Canadians who underwent elective total joint arthroplasty (TJA) surgery in 2012–2013 for hip and knee osteoarthritis (OA), 40 and 60 %, respectively, were obese. Obesity is associated with increased risks for receiving TJA, post-operative complications and delayed functional recovery. Current guidelines for patients with a body mass index (BMI) of ≥30 kg/m^2^ are to participate in a weight management programme and to lose weight prior to TJA surgery. As part of a larger project, a rapid review was conducted to examine the effects of short-term non-pharmacological and non-surgical weight loss interventions in adults in the year prior to total hip arthroplasty (THA) and total knee arthroplasty (TKA) on surgical and patient outcomes, and adverse events.

**Methods:**

We performed a rapid review and searched seven electronic databases for English language articles published between 1990 and February 2015. Observational studies evaluating the association between pre-operative weight loss and short- and long-term outcomes, and controlled trials of non-pharmacological and non-surgical weight loss interventions were considered for inclusion. Two reviewers independently screened and selected articles, assessed methodological quality and extracted data.

**Results:**

Of 263 articles identified, a total of four studies met our inclusion criteria. In one of two high-quality retrospective cohort studies, weight loss ≥5 % of body weight in the year prior to TJA and maintained in the year after surgery was associated with a higher likelihood of deep surgical site infection in THA patients and 90-day readmission in TKA patients. No significant differences were reported in incidence of superficial surgical site infections in THA or TKA patients who lost weight pre-operatively compared to those who maintained their weight in either study. Two abstracts of randomized controlled trials were included; however, despite contacting the authors, full-length articles were not available. The limited information from the trials suggested that short-term dietician-supervised weight loss interventions were effective in weight loss prior to TJA.

**Conclusions:**

There is limited evidence to support the recommendation of weight loss in the year prior to TJA and to determine the effectiveness of short-term non-pharmacological, non-surgical weight management interventions on patient and surgical outcomes.

## Background

Obesity, defined as having a body mass index (BMI) of ≥30 kg/m^2^ [1], is strongly associated with the development of osteoarthritis (OA) of the knee [2, 3] and to a lesser extent of the hip joint [4]. Obesity is also a risk factor for OA progression [5] and for receiving a total joint arthroplasty (TJA) [6, 7]. Of the more than 104,000 TJA procedures performed in Canada in 2012–2013, 40 % of total hip arthroplasty (THA) and 60 % of total knee arthroplasty (TKA) patients were obese (classes I, II or III based on 2011–2012 data) [8]. Candidates for TJA who are obese are often advised to lose weight prior to surgery as obesity is associated with increased peri- and post-operative complications [9] and slower functional improvement post-operatively [10–13]. The literature suggests that obesity is one comorbidity that increases the risk of complications such as infection, thromboembolic events and long-term prosthetic survivorship [9, 14]. Obesity is also associated with longer hospital stays and higher costs in TKA [15]. From a surgeon’s perspective, obesity presents additional challenges with increased risk of component malposition and prosthesis loosening and dislocation [14]. Because obesity is a risk factor for diabetes mellitus, coronary artery disease, hyperlipidemia, hypertension and sleep apnoea, post-operative complications associated with obesity are typically confounded by other chronic conditions [11]. Nonetheless, the current recommendations based on international guidelines are to acknowledge and mitigate the risks associated with obesity in patients undergoing TJA surgery [16, 17]. In a more direct approach, several international organizations include in their online and print patient education materials the recommendation to lose weight if suggested by a physician [18–20]. The most recent of the two guidelines included papers published before April 2011, and therefore, more recent evidence may provide further guidance on this topic.

The aim of this rapid review was to examine the evidence to support the recommendation to lose weight prior to elective TJA and to inform the development of a quality indicator (QI) on weight management in the year prior to surgery. The research question asked was what are the effects of short-term non-pharmacological and non-surgical weight loss interventions on pre- and post-operative TJA outcomes? In contrast, pharmacological interventions may include prescribed and over-the-counter products (e.g. bupropion and orlistat) and surgical interventions would include different forms of bariatric surgery [21]. A rapid review approach was chosen over a systematic review methodology in order to synthesize the evidence in a timely, less resource-intensive manner so that findings may be aligned with the timeline for the larger project addressing QIs for TJA rehabilitation. While not defined by a single methodology, rapid reviews can incorporate some key elements of systematic reviews including comprehensive search strategies, dual study selection and data extraction, quality assessment and transparency in reporting to increase their rigour and reliability of findings [22].

## Methods

We included randomized controlled trials (RCTs), controlled trials and cohort (prospective and retrospective) studies with a control or comparison group. We did not include case series, single-subject, cross-sectional or qualitative designs. Using the PICO framework to identify patient/population, intervention(s), control/comparator and outcomes, we specified *a priori* the following inclusion criteria: (1) patients with hip or knee OA scheduled for or awaiting primary TJA who were obese (BMI ≥30 kg/m^2^); (2) non-pharmacological, non-surgical weight management interventions including one-to-one or group counselling, education, dietary, cognitive behavioural and exercise interventions delivered by health professional(s) alone or in combination provided within 1 year of TJA surgery; (3) studies with a control group in which participants received no additional advice to lose weight, information only about weight management (e.g. brochure, handout), or participated in an unrelated educational programme (attention control); and (4) studies reporting on one or more of the following outcomes—pain, self-reported or performance-based function, quality of life, surgery-related post-operative complications (e.g. surgical site infection), other adverse events, hospital readmission rate (e.g. 30 days) and hospital length of stay (LOS).

*Search strategy*. We performed an electronic search of Medline, EMBASE, CINAHL, Cochrane Central Register of Controlled Trials (CENTRAL), Psych Info, PEDro and OT Seeker databases for relevant articles using the following MeSH terms and key words: “hip replacement”, “hip arthroplasty”, “knee replacement”, “knee arthroplasty” in combination with the terms “obesity”, “weight reduction”, “weight management”, “weight loss” and “bariatrics”. Reference lists of retrieved articles and relevant practice guidelines were scanned for additional papers. The only limits applied to the search were that studies had to be published in English between January 1990 and February 24, 2015 to align with the larger QI project (see Table 1 for Medline search strategy).

**Table 1:** Medline search strategy

Number	Searches
1	Hip Prosthesis/ or Arthroplasty, Replacement, Hip/
2	((hip adj3 arthroplast*) or (hip adj3 replace*)).mp.
3	1 or 2
4	Knee Prosthesis/ or Arthroplasty, Replacement, Knee/
5	((knee adj3 arthroplast*) or (knee adj3 replace*)).mp.
6	4 or 5
7	Obesity/dh, th, rh [Diet Therapy, Therapy, Rehabilitation]
8	Weight Loss/
9	Bariatrics/
10	((weight adj3 loss) or (weight adj3 manage*) or (weight adj3 reduc*)).mp.
11	7 or 8 or 9 or 10
12	3 or 6
13	11 and 12
14	limit 13 to (English language and humans and yr=”1990-current”)

Initial screening by title and abstract and full-text screening were performed independently by two reviewers (ML and MW). Any disagreements were resolved by consensus. Reviewers independently extracted patient demographic, control, outcome and variable data from included papers and verified results with each other to ensure consensus. The same two reviewers assessed the methodological quality of cohort studies using the Newcastle Ottawa Scale (NOS), a tool developed to assess the quality of non-randomized studies with respect to three areas: selection of the study groups, the comparability of the groups and the ascertainment of either the exposure or outcome of interest [23]. We planned to assess controlled trials using the Cochrane Risk of Bias tool [24]; however, no full-length controlled intervention studies were identified.

The methodological approaches used in our rapid review differ from the traditional systematic review in that we used a less comprehensive search strategy (i.e. limited grey-literature search, no attempt to contact leading authors in the field, inclusion of English-only articles), did not assess for possible publication bias (i.e. creation of funnel plots) and made no plan to pool the data and perform a meta-analysis if warranted by clinical and methodological homogeneity among studies.

## Results

**Fig. 1:**
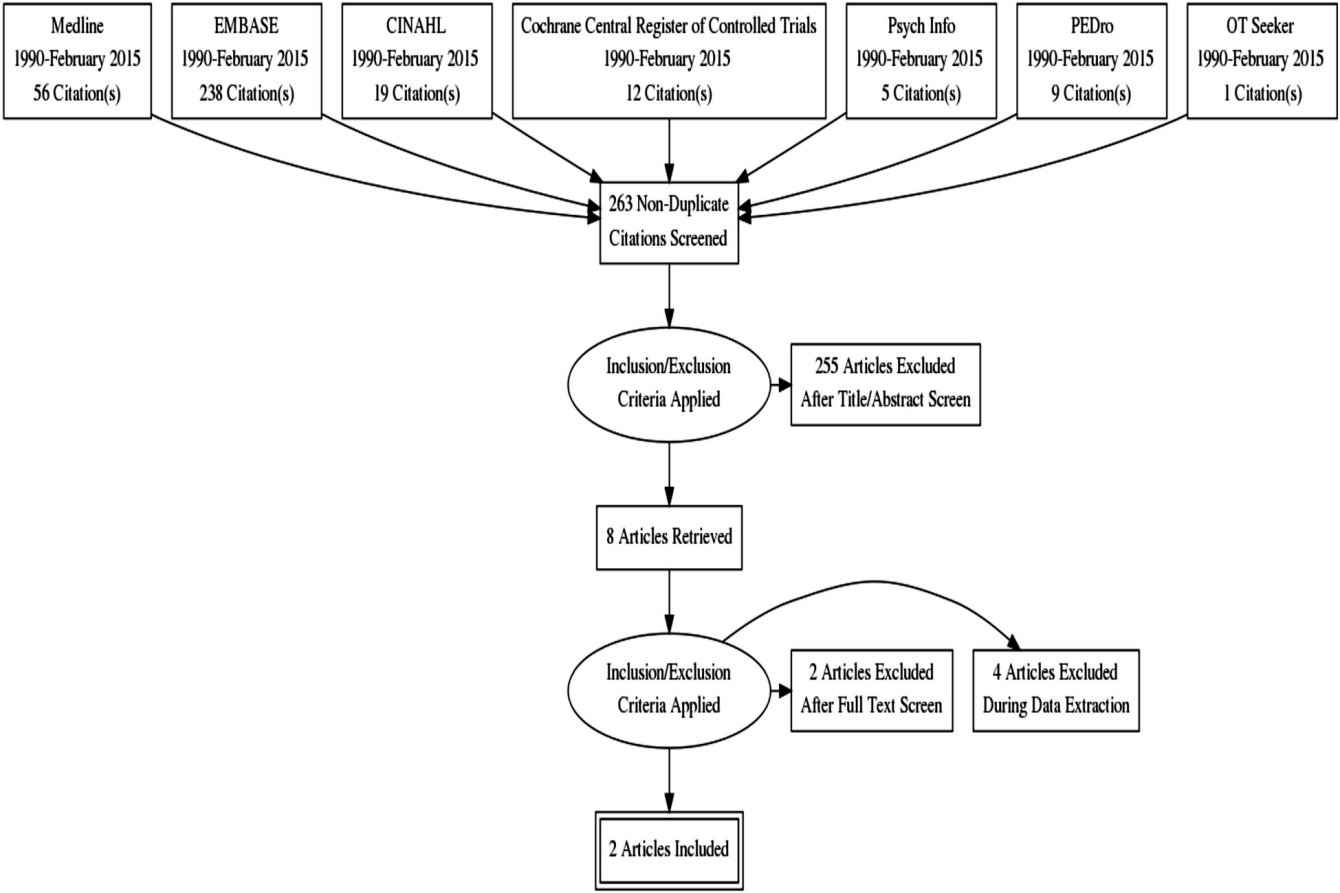
PRISMA flow diagram

Our search identified 263 articles and abstracts published in English between 1990 and February 24, 2015. Of these, eight records passed the initial screening of the title and abstract review (six full-length articles and two abstracts) and were assessed for eligibility (see Fig. 1) [9, 25–31]. A total of four records met the inclusion criteria (two cohort studies, two RCT abstracts). The two intervention studies were only available in abstract format at the time of the review [30, 31]. We contacted the authors of these abstracts to determine if a full-length article had since been published. In both cases, the papers were in pre-publication status and therefore not available for review and methodological assessment. We have addressed each briefly to inform the discussion on this topic.

There is limited research on the association of weight loss prior to TJA and effectiveness of short-term non-pharmacological and non-surgical weight loss interventions prior to TJA surgery on pre- and post-operative outcomes and adverse events. Two retrospective cohort studies by the same authors, Inacio and colleagues, examined the association between pre-operative weight loss and peri-operative and post-operative outcomes [26, 27] (see Table 1 for study descriptions and results). These studies used different samples of data from the Total Joint Replacement Registry in California and electronic health records of patients in a large integrated healthcare organization/system. Patients who were identified as obese (BMI ≥30 kg/m^2^) between January 1, 2008 and December 31, 2010 and scheduled for primary unilateral TKA or THA for OA with no history of surgical weight loss intervention were included. The authors considered a decrease of ≥5 % of body weight as clinically significant weight loss. It is important to note that the methods patients used to lose weight were not available through the registry data; however, patients undergoing bariatric surgery were excluded.

In the first cohort study of 9675 patients, 14.3 % of the patients lost ≥5 % of their body weight in the year prior to surgery with more females (15.8 %) losing weight than males (11.9 %) [26]. Mean weight loss and intraoperative BMI were not reported. However, a greater proportion of patients who lost weight were in the <30 kg/m^2^ BMI category at the time of surgery (27 versus 5.1 %) suggesting successful weight loss or that those patients with lower starting BMI levels pre-operatively were more successful in losing additional weight [26]. After adjusting for age and comorbidity of congestive heart failure, the authors found that there was a 63 % greater likelihood of 90-day readmission [odds ratio (OR) 1.63 (95 % confidence interval (CI) 1.16–2.28)] in patients undergoing TKA who lost weight in the year prior to surgery and kept it off compared with those who remained the same weight for the entire study period [26]. No significant differences in the likelihood of surgical site infections were found over 12 months of follow-up. In obese patients undergoing THA, after adjusting for intraoperative BMI, patients who lost weight and kept it off in the year after surgery were greater than three times more likely [OR 3.77 (95 % CI 1.59–8.95)] to develop a deep surgical site infection than those who remained the same weight.

In the second cohort study of 14,784 patients, 14.0 % of the patients lost ≥5 % of their body weight in the year prior to TJA surgery (THA 18.0 %, TKA 12.4 %) while another 7.3 % gained weight and 78.3 % remained the same weight during this period. After adjusting for the covariates gender, age, intraoperative BMI, blood loss and congestive heart failure, the risks of deep and superficial surgical site infections and 90-day readmission were not significantly different in the patients who gained or lost ≥5 % of their body weight compared to those with no weight change in either the TKA or THA sample [27] (see Table 2 for study description and results).

**Table 2:** Study description and results for cohort studies

Study, design and quality	Demographics				Outcomes and results		
Inacio et al. [26]		Total	THA	TKA		THA	TKA
USA	No. of participants	9675	2554	7121	DSSI (adjusted OR and 95 % CI)	3.77 (1.59–8.95)	1.67 (0.77–3.61)
Retrospective cohort	Lost ≥5 % of body weight (%)^a^	1381 (14.3)	444 (17.4)	937 (13.2)	SSSI (adjusted OR and 95 % CI)	0.95 (0.27–3.30)	1.41 (0.41–4.85)
NOS quality score: 9	Gender (% female) who:				90-day Re-ad (adjusted OR and 95 % CI)	1.18 (0.72–1.93)	1.63 (1.16–2.28)
	• Lost weight	67	65.1	68.2			
	• Stayed the same	59.7	52.6	62.1			
	Age (% <65 years)^b^	43.9	46.4	43.0			
	BMI (%)^b^						
	• ≥30 and <35 kg/m^2^	52.1	55.2	51.0			
	• ≥35 kg/m^2^	39.7	33.3	41.9			
ᅟ							
Inacio et al. [27]		Total	THA	TKA		THA	TKA
USA	No. of participants	14,784	4066	10,718	DSSI (adjusted OR and 95 % CI)	1.83 (0.83–4.02)	1.27 (0.66–2.42)
Retrospective cohort	Lost ≥5 % of body weight (%)^a^	2064 (14.0)	732 (18.0)	1332 (12.4)	SSSI (adjusted OR and 95 % CI)	1.16 (0.43–3.13)	0.83 (0.29–2.37)
NOS quality score: 9	Gender (% female) who:				90-day Re-ad (adjusted OR and 95 % CI)	1.05 (0.70–1.57)	1.20 (0.88–1.63)
	• Lost weight	64.9	61.2	67			
	• Stayed the same	60.7	61.2	63.4			
	• Gained weight	65.3	54.3	68.8			
	Age (% <65 years)^b^	46.6	50.8	33.0			
	BMI (%)^b^						
	• ≥30 and <35 kg/m^2^	50.8	53.5	49.7			
	• ≥35 kg/m^2^	41.0	35.3	43.2			

*DSSI* deep surgical site infection up to 1 year post-TJA, *SSSI* superficial surgical site infection within 30 days of TJA, *Re-ad* readmission, *NOS* Newcastle Ottawa Scale for cohort studies

^a^Number of patients who lost weight pre-operatively and kept it off post-operatively

^b^Percentage based on total sample size

Abstracts of the two intervention studies were retrieved; however, full-length articles were not available to inform this rapid review. In a pilot RCT, Simmance and colleagues randomly assigned 40 patients with BMI ≥30 kg/m^2^ to either a weight loss intervention consisting of at least four in-person or telephone-based sessions with a licensed dietician or usual care consisting of healthy eating advice by a preadmission clinic nurse [30]. The primary outcomes were weight loss and improvement in self-reported physical health scores at 12 months after THA or TKA. Results indicated that a structured dietician-led weight loss intervention prior to TJA is more effective in achieving weight loss than usual care and resulted in a statistically significant improvement in self-reported physical health 12 months after surgery. No adverse events were reported in the abstract.

As part of a RCT, researchers initially examined the safety and feasibility of implementing an intensive weight loss programme before surgery in order to reduce pre-operative weight in patients undergoing TKA [31]. Thirty-eight patients scheduled for TKA with a BMI >30 kg/m^2^ and identified as motivated to lose weight were included in the intervention group of the RCT. Patients followed a dietician-supervised 8-week weight loss programme consisting of nutritional education and a low-calorie diet (810 kcal/day) using commercially available formula foods pre-operatively. Adverse events included dry skin in three patients, constipation in four and postponement of TKA surgery in one 70-year-old male patient due to cardiac arrhythmia. However, the treating physicians reported none of the episodes was related to the weight loss intervention. All patients completed the intervention; however, results for the outcomes identified in the published protocol for this trial were not included in the abstract. The authors concluded that it was safe to implement a weight loss programme shortly before TKA.

## Discussion

Current international guidelines universally recommend that obese patients with hip and knee OA lose weight to reduce arthritis symptoms [32–35] and identify and mitigate risks associated with obesity prior to undergoing elective TJA [16, 17]. Furthermore, there is a growing body of evidence demonstrating a negative but inconsistent association of obesity with peri-operative and post-TJA outcomes [10–14, 36, 37]. However, we were unable to find conclusive evidence to support the recommendation that obese patients lose weight prior to TJA.

In both cohort studies, the authors highlighted important considerations to explain their research findings including the possibility of residual confounding factors from unevaluated characteristics, unintended consequences of weight loss (i.e. malnutrition) which can be associated with poor outcomes after TJA, and post-operative metabolic stress (catabolic state or hyperglycemia) induced by trauma of the surgical procedure which could in turn increase the patient’s risk for complications. Malnutrition has been associated with serious complications in patients undergoing elective TJA [38] including increasing the risk of surgical site infection [39]. Of note, findings from a prospective cohort study of patients undergoing elective TJA surgery revealed that 42.9 % of malnourished patients in the cohort were obese and had a significantly higher post-operative complication rate [38]. Findings from other patient populations such as hip fracture suggest that low serum albumin indicative of malnutrition is associated with higher mortality and complication rates [40].

### Clinical implications

Healthcare providers have an important role to play in counselling patients prior to elective TJA surgery. Weight loss within the year prior to TJA surgery is often challenging for patients who are overweight as increasing overall physical activity or participating in an exercise programme is difficult with advanced hip or knee OA and associated pain, impairment and activity limitations. Current data from two high-quality retrospective cohort studies suggest increased risk of deep surgical site infection and 90-day hospital readmission with weight loss of ≥5 % over the year prior to TJA with the caveat that no information was provided on how weight loss was achieved and/or whether there were nutritional implications or malnutrition that could have compromised healing. Considering the paucity of evidence and the potential to increase the risk of surgical site infection with weight loss of ≥5 % of body weight prior to TJA in patients who are obese, the question is raised around the safety and clinical effectiveness of this recommendation. Further research is warranted on this topic to better inform patients on the risks and benefits of pre-TJA weight loss. Specifically, future studies should address the safety (adverse events), optimal timing (how much prior to TJA surgery), duration (length of weight management programme), amount (percentage of body weight) and the most effective non-surgical/non-pharmacological interventions (dietary, exercise, educational, behavioural) and delivery methods (in person, telephone, web-based) for pre-TJA weight loss in a prospective and controlled fashion.

Strengths of this rapid review include efforts to reduce bias by having two independent reviewers screen, select and extract data and evaluating study quality through the use of a standardized tool for cohort studies. Seven electronic databases were searched, and all the authors were contacted for further information to ensure clarity and accuracy. Both reviewers have clinical expertise with this patient population. Limitations internal to rapid reviews include limiting our search to published English language articles and thus likely missing unpublished reports (i.e. grey literature) and those papers published in other languages that may have informed this topic. Had our timeline permitted a more rigorous and comprehensive systematic review on this and the numerous other topics addressed by the larger quality indicators project, it is possible that other evidence may have been available to us. Secondly, the lack of access to the full-length articles of the two intervention studies limited our ability to determine the quality and strength of this evidence and prevented an in-depth examination of their effectiveness.

## Conclusions

To date, there is insufficient evidence to support the recommendation that patients who are obese lose weight (≥5 %) within the year prior to either THA or TKA. The available cohort studies retrospectively examined weight patterns before and after TJA in patients who were obese but did not specify the weight loss method. Two abstracts of dietician-supervised weight loss interventions show promising results; however, given the fact that full-length papers were not available, study methodology could not be assessed and data could not be extracted. The limited evidence found through this rapid review suggests that this is a topic for further research considering the prevalence of obesity in patients undergoing TJA surgery.

## Abbreviations

BMI: body mass index
CI: confidence interval
LOS: length of stay
NOS: Newcastle Ottawa Scale
OA: osteoarthritis
OR: odds ratio
QI: quality indicator
RCT: randomized control trial
THA: total hip arthroplasty
TJA: total joint arthroplasty
TKA: total knee arthroplasty
